# Artificial intelligence literacy and readiness in future health care professionals: a cross-sectional study

**DOI:** 10.3325/cmj.2026.67.4

**Published:** 2026-02

**Authors:** Funda Catan-Inan

**Affiliations:** Department of Medicine-Biostatistics, Bilecik Seyh Edebali University, Bilecik, Turkey

## Abstract

**Aim:**

To examine the relationship between artificial intelligence (AI) literacy and readiness for medical AI among medical and nursing students, and to explore demographic and educational factors affecting AI literacy and readiness for AI.

**Methods:**

This cross-sectional study enrolled 443 students attending the Faculty of Medicine and the Department of Nursing at Bilecik Seyh Edebali University between May and June 2025. Data were collected using a general demographic questionnaire, Medical Artificial Intelligence Readiness Scale (MAIRS), and Artificial Intelligence Literacy Scale (AILS).

**Results:**

Medical students showed higher AI readiness (*P* < 0.001) and literacy (*P* = 0.032) scores than nursing students. AI literacy was moderately and positively correlated with readiness for medical AI (r = 0.338, *P* < 0.001), and the associations were strongest on the Awareness and Usage dimensions. In multivariable models, MAIRS scores were independently associated with the study year, department, and single-item readiness to use AI, while AILS scores were independently associated with the readiness to use AI and the belief that AI could partially replace health care workers. Internal consistency of the questionnaires was high (MAIRS α = 0.972; AILS α = 0.878).

**Conclusion:**

The findings support the integration of structured, practical, and ethically informed AI education into health science curricula, as this may be associated with greater student readiness for future AI-supported health care environments.

Artificial intelligence (AI) has considerably transformed the health care landscape, with applications ranging from clinical decision support and disease diagnosis to personalized treatment planning. AI-supported clinical decision-making tools have been shown to enhance diagnostic accuracy, reduce health care professionals’ workload, and improve overall patient care outcomes (1). As these technologies become increasingly integrated into clinical practice, the awareness and readiness of future health care professionals – particularly medical and nursing students – emerge as critical factors for the effective and ethical deployment of AI in health care settings (2-5). In this context, investigating the relationship between AI literacy and readiness among health care students offers valuable insights into how to integrate AI into future health care delivery systems.

AI literacy is a multifaceted construct encompassing individuals’ foundational knowledge of AI technologies, their ability to effectively use these tools, ethical awareness, and capacity for critical evaluation. The level of AI literacy determines the ability of health care professionals to comprehend, implement, and critically assess AI-supported systems within clinical settings. Closely related, yet distinct, readiness for medical AI refers to individuals’ perceptions, acceptance levels, and willingness to integrate AI technologies into health care practice. Together, these dimensions provide a framework for understanding how future health care providers may engage with the evolving landscape of AI in medicine.

Although recent studies show a growing awareness of AI among medical and nursing students, there remain considerable gaps in their practical knowledge and skills (6). Early exposure to AI-related content has been shown to positively influence students’ readiness to engage with AI-based systems (7). However, there is a lack of studies that directly examine the relationship between AI literacy and readiness for medical AI. The results of this study may help us understand how educational interventions can better prepare future health care professionals for an AI-supported clinical environment.

This study investigated the relationship between AI literacy and readiness for medical AI among medical and nursing students. The secondary aim was to evaluate the influence of students’ demographic and descriptive characteristics on their levels of AI literacy and readiness.

## Participants and methods

This cross-sectional study enrolled 443 students attending the Faculty of Medicine or the Department of Nursing, Faculty of Health Sciences at Bilecik Seyh Edebali University from May to June 2025. The inclusion criteria were being an undergraduate medical or nursing student and being ≥18 years old. The exclusion criterion was not completely responding to the survey questions. The sample was selected using a convenience sampling method. Sample size calculation was performed using G*Power version 3.1. Based on a two-tailed alpha error of 0.05, a power of 0.95, and an effect size (Cohen’s d) of 0.4, the minimum sample size required to achieve statistical power for the independent-samples *t* test was 328 participants.

### Instruments

*Student general demographic questionnaire.* A general demographic questionnaire was developed by the researcher based on a comprehensive review of the literature and was used to collect data on students’ gender, age, department of study, academic year, grade point average (GPA), duration of internet use, use of AI, purposes for using AI, and opinions regarding the future role of AI in health care.

*Artificial Intelligence Literacy Scale (AILS).* The AILS was developed to measure individuals’ knowledge of AI technologies, usage skills, ethical awareness, and critical thinking capacities (8). Adapted into Turkish by Çelebi et al (9), the scale comprises 12 items organized into four subdimensions: Awareness, Usage, Evaluation, and Ethics. The questions are answered on a seven-point Likert scale (1: strongly disagree – 7: strongly agree), and the score ranges from 12 to 84, with higher scores indicating a higher level of AI literacy (9). Items 2, 5, and 11 are reverse scored. The scale demonstrated high internal consistency, with the Cronbach’s alpha coefficient of 0.85 for the overall original scale and subdimensions: 0.83 (total), 0.73 (Awareness), 0.75 (Usage), 0.78 (Evaluation), and 0.73 (Ethics) (8). The internal consistency values of the Turkish adaptation were 0.85 (total), 0.72 (Awareness), 0.74 (Usage), 0.76 (Evaluation), and 0.72 (Ethics) (9).

*Medical Artificial Intelligence Readiness Scale (MAIRS).* The MAIRS was developed by Karaca et al to evaluate the extent to which students of medicine and health sciences are cognitively and ethically prepared to engage with AI technologies in clinical and academic settings (10). The instrument comprises 22 items across four subscales: Cognition, Ability, Vision, and Ethics. Responses are recorded on a five-point Likert scale ranging from 1 (strongly disagree) to 5 (strongly agree), yielding a total score between 22 and 110. Higher scores indicate greater readiness to interact with medical AI. The internal consistency of the scale, as measured by Cronbach’s alpha, was 0.87, indicating a high level of reliability.

### Data collection

The survey was developed using Google Forms. A statement at the beginning of the questionnaire explained the purpose of the study and informed the respondents that the participation was voluntary and that they could withdraw their consent immediately following the initial question.

Participants who completed and submitted the online survey were considered to have given their informed consent to participate in the study. To ensure data integrity, the survey was restricted to a single submission per IP address. To guarantee complete responses, the “Required” option was enabled for all items; consequently, respondents who left any questions unanswered were automatically excluded from the study. The survey remained open for one month. Respondents completed the questionnaire anonymously. Data were collected by the researcher through face-to-face meetings with students in their classrooms during pre-class sessions via an online platform from May to June 2025. The study complied with all the principles of the Declaration of Helsinki. It was approved by the Ethics Committee of Bilecik Seyh Edebali University, Faculty of Medicine.

### Statistical analysis

Participants’ characteristics are summarized using descriptive statistics, including frequencies, percentages, means, standard deviations, and minimum-maximum values. The normality of data distribution was assessed using kurtosis and skewness values. Since both values between +1.5 and −1.5 indicate a normal distribution, all sub-dimensions of the two scales and the total score of the scales showed a normal distribution (11). The Pearson χ^2^ test was used to assess the differences between categorical variables. The independent-samples *t* tests and one-way ANOVA with a *post-hoc* Tukey comparison test were performed to assess the differences between continuous variables. Effect sizes were reported as Cramer’s V for the Pearson χ^2^ test, Cohen’s d for *t* tests, and partial eta squared (ηp^2^) for ANOVA. Multivariable linear regression analysis was performed to identify factors associated with MAIRS and AILS scores, with categorical variables entered into the models using dummy coding and the first category defined as the reference group. The correlations between AI literacy and readiness for medical AI was evaluated using Pearson correlation coefficient. All statistical tests were two-tailed, and the results were interpreted at a 95% confidence interval with a significance threshold set at *P* < 0.05. Statistical analysis was conducted using SPSS, version 26.0 (IBM Corp., Armonk, NY, USA).

## Results

A total of 443 participants were included in the study, of whom 285 (64.3%) were women. The mean age was 20.67 ± 1.96 years. The average academic grade point average (GPA) was 2.86 ± 0.22, graded on a four-point scale (range 1.00-4.00), where higher scores indicate better academic performance.

Discipline was significantly associated with gender (*P* < 0.001; Cramer’s V = 0.313), with nursing students being predominantly female and medical students more evenly distributed by gender. The department was also significantly associated with the study year (*P* < 0.001; V = 0.273), with medical students mainly being in the first year, while nursing students were more dispersed across the study years. Nursing students used the internet for significantly longer periods than medical students (*P* = 0.009; V = 0.146). AI course attendance was more common among medical students (*P* = 0.002; V = 0.151), which may be linked to their higher levels of readiness to use AI (*P* = 0.008; V = 0.176). Medical students were more likely to use AI for daily information seeking and nursing students for academic research (*P* = 0.045; V = 0.135). Nursing students more frequently reported moderate or good knowledge regarding AI than other knowledge levels, while medical students were distributed more widely across the spectrum (*P* = 0.020; V = 0.162). In contrast, the groups did not significantly differ in their perceptions of AI’s future role in health care (*P* = 0.821; V = 0.030) and of AI replacing health care workers (*P* = 0.769; V = 0.034). Overall, effect sizes ranged from negligible to moderate, with the largest associations observed for gender and study year (Table 1).

**Table 1:** Demographic and artificial intelligence (AI)-related characteristics of medical and nursing students

	Total n (%)	Medicine n (%)	Nursing n (%)	*X^2^*	*P*	Cramer’s V
**Gender**						
female	285 (64.3)	85 (46.4)	200 (76.9)	43.47	<0.001	0.313
male	158 (35.7)	98 (53.6)	60 (23.11)			
**Study year**						
first	164 (37.0)	86 (47.0)	78 (30.0)	33.09	<0.001	0.273
second	145 (32.7)	64 (35.0)	81 (31.2)			
third	106 (23.9)	33 (18.0)	73 (28.1)			
fourth	28 (6.3)	0 (0)	28 (10.8)			
**Daily internet usage time (hours)**						
<3	139 (31.4)	72 (39.3)	67 (25.8)	9.45	0.009	0.146
4-6	227 (51.2)	81 (44.3)	146 (56.2)			
>7	77 (17.4)	30 (16.4)	47 (18.1)			
**Previous AI course**						
yes	68 (15.3)	40 (21.9)	28 (10.8)	10.16	0.002	0.151
no	375 (84.7)	143 (78.1)	232 (89.2)			
**Interest in AI technologies**						
yes	215 (48.5)	92 (50.3)	123 (47.3)	1.07	0.586	0.049
no	44 (9.9)	20 (10.9)	24 (9.2)			
partial	184 (41.5)	71 (38.8)	113 (43.5)			
**Readiness to use AI**						
do not plan to use AI	25 (5.6)	14 (7.7)	11 (4.2)	13.67	0.008	0.176
willing but not ready	51 (11.5)	21 (11.5)	30 (11.5)			
need training	181 (40.9)	63 (34.4)	118 (45.4)			
currently use some AI systems	117 (26.4)	45 (24.6)	72 (27.7)			
actively use and open to development	69 (15.6)	40 (21.9)	29 (11.2)			
**AI use purpose**						
academic research	221 (49.9)	80 (43.7)	141 (54.2)	8.05	0.045	0.135
daily information seeking	141 (31.8)	68 (37.2)	73 (28.1)			
entertainment/general use	52 (11.7)	26 (14.2)	26 (10.0)			
have never used AI	29 (6.5)	9 (4.9)	20 (7.7)			
**Knowledge of AI in health care**						
no	27 (6.1)	15 (8.2)	12 (4.6)	11.63	0.020	0.162
low	89 (20.1)	45 (24.6)	44 (16.9)			
moderate	206 (46.5)	78 (42.6)	128 (49.2)			
good level	98 (22.1)	32 (17.5)	66 (25.4)			
very good level	23 (5.2)	13 (7.1)	10 (3.8)			
**AI hasfuture role in health care**						
agree	342 (77.2)	144 (78.7)	198 (76.2)	0.39	0.821	0.030
neutral	80 (18.1)	31 (16.9)	49 (18.8)			
disagree	21 (4.7)	8 (4.4)	13 (5.0)			
**AI will replace health care workers**						
yes	57 (12.9)	24 (13.1)	33 (12.7)	0.526	0.769	0.034
no	205 (46.3)	81 (44.3)	124 (47.7)			
partly	181 (40.9)	78 (42.6)	103 (39.6)			

Significantly higher scores on both MAIRS (*P* = 0.001) and AILS (*P* = 0.011) were reported by men compared with women and by medical students compared with nursing students (*P* < 0.001 and *P* = 0.032, respectively). MAIRS scores were significantly higher among second-year and third-year students compared with first-year students (*P* = 0.001) and among students who had taken an AI-related course compared with those who had not (*P* = 0.048). MAIRS (*P* < 0.001, Tukey test) and AILS (*P* < 0.001, Tukey test) scores were significantly higher among students interested in AI and among those actively using and open to developing AI systems compared with the other groups. MAIRS scores (*P* = 0.001) were higher in those using AI for daily information seeking or academic research. Students with no knowledge had significantly lower MAIRS scores than all other knowledge groups (*P* = 0.012). MAIRS and AILS scores (*P* = 0.005 and *P* < 0.001, respectively) were higher among those who agreed that AI had a future role in health care than in those who disagreed. Lastly, AILS (*P* < 0.001) and MAIRS scores were significantly higher in respondents who believed that AI would partly replace health care workers compared with those who answered “yes” or “no”. Although several comparisons were statistically significant, the corresponding effect sizes were generally small, indicating modest differences between the groups (Table 2).

**Table 2:** Medical Artificial Intelligence Readiness Scale (MAIRS) and Artificial Intelligence Literacy Scale (AILS) scores according to socio-demographic characteristics

	MAIRS X̄±SD	test	*P*	Effect size	AILS X̄ ±SD	test		*P*	Effect size
**Gender**									
female	68.23 ± 16.86	−3.45	0.001	0.37	58.72 ± 10.19	−2.55		0.011	0.27
male	75.34 ± 22.65				61.85 ± 13.40				
**Department**									
medicine	75.09 ± 19.53	4.00	<0.001	0.39	61.28 ± 12.83	2.15		0.032	0.21
nursing	67.72 ± 18.76				58.82 ± 10.41				
**Study year**									
first	66.53 ± 16.78	5.66	0.001	0.037	58.06 ± 9.25	2.38		0.068	0.016
second	75.25 ± 20.58				61.49 ± 13.73				
third	71.90 ± 20.08				60.08 ± 12.24				
fourth	68.00 ± 20.20				60.75 ± 5.86				
**Daily internet usage time (hours)**									
<3	73.07 ± 21.10	1.68	0.186	0.008	60.89 ± 13.11	1.20		0.300	0.005
4-6	70.17 ± 17.39				59.69 ± 9.64				
>7	68.35 ± 21.56				58.38 ± 13.38				
**Previous AI course**									
yes	76.11 ± 24.78	2.01	0.048	0.33	61.98 ± 14.87	1.34		0.184	0.22
no	69.79 ± 18.13				59.45 ± 10.79				
**Interest in AI technologies**									
yes	74.58 ± 21.41	9.05	<0.001	0.040	62.00 ± 13.05	7.75		<0.001	0.034
no	64.13 ± 17.06				56.88 ± 8.08				
partial	67.89 ± 16.41				58.02 ± 9.76				
**Readiness to use AI**									
do not plan	61.00 ± 14.65	14.05	<0.001	0.114	59.92 ± 8.15	10.85		<0.001	0.090
willing but not ready	63.74 ± 16.89				55.33 ± 9.23				
need training	67.80 ± 16.31				58.36 ± 8.72				
currently use some systems	72.71 ± 17.85				60.45 ± 11.65				
actively use and open to development	83.95 ± 25.22				67.07 ± 16.13				
**AI use purpose**									
academic research	70.57 ± 18.63	5.37	0.001	0.035	59.08 ± 11.16		2.49	0.059	0.017
daily information seeking	74.63 ± 20.71				61.92 ± 12.56				
entertainment/general use	66.63 ± 18.75				58.88 ± 11.64				
have never used AI	60.79 ± 14.82				57.20 ± 6.84				
**Knowledge of AI in health care**									
no	63.62 ± 17.69	3.23	0.012	0.029	59.48 ± 10.50		1.94	0.104	0.017
low	67.76 ± 15.42				58.01 ± 9.26				
moderate	70.32 ± 18.92				59.51 ± 11.09				
good	75.01 ± 20.62				61.17 ± 13.23				
very good	76.65 ± 27.39				64.60 ± 15.19				
**AI has future role in health care**									
agree	72.41 ± 19.73	5.47	0.005	0.024	61.06 ± 11.64		9.09	<0.001	0.040
neutral	65.37 ± 16.86				56.26 ± 9.48				
disagree	64.61 ± 18.94				53.66 ± 12.27				
**AI will replace health care workers**									
yes	65.43 ± 20.94	3.03	0.049	0.014	57.05 ± 12.46		7.89	<0.001	0.035
no	70.59 ± 16.04				58.38 ± 10.77				
partly	72.64 ± 22.00				62.37 ± 11.62				

In multivariable linear regression analysis, several factors were independently associated with MAIRS scores (Table 3): second study year vs first study year (*B* = 5.94, *P* = 0.005), the study of medicine vs the study of nursing (*B* = −5.55, *P* = 0.005), currently using some AI systems (*B* = 10.41, *P* = 0.017), and actively using AI systems and being open to further development (*B* = 17.86, *P* < 0.001). The factors associated with AILS were single-item readiness to use AI (*B* = 7.60, *P* = 0.009) and believing that AI could partly replace health care workers (*B* = 3.82, *P* = 0.026). Other variables, including gender, daily internet use time, prior AI education, and interest in AI technologies, were not independently associated with MAIRS or AILS scores.

**Table 3:** Multivariable linear regression analysis of factors associated with Medical Artificial Intelligence Readiness Scale (MAIRS) and Artificial Intelligence Literacy Scale (AILS) scores

	MAIRS					AILS				
Predictor	B (SE)	95% CI	β	t	*P*	B (SE)	95% CI	β	t	*P*
**Intercept**	61.49 (5.46)	50.76-72.23		11.26	<0.001	55.45 (3.35)	48.87-62.03		16.56	<0.001
**Gender**										
female-male	2.64 (1.99)	−1.26-6.55	0.14	1.33	0.185	0.47 (1.22)	−1.93-2.86	0.04	0.38	0.701
**Class level**										
second-first	5.94 (2.12)	1.77-10.10	0.31	2.80	0.005	1.97 (1.30)	−0.58-4.52	0.17	1.52	0.130
third-first	3.46 (2.36)	−1.17-8.09	0.18	1.47	0.143	1.26 (1.45)	−1.58-4.10	0.11	0.87	0.385
fourth-first	2.67 (3.89)	−4.97-10.33	0.14	0.69	0.492	3.60 (2.39)	−1.08-8.29	0.31	1.51	0.132
**Department**										
nursing-medicine	−5.55 (1.95)	−9.39- −1.72	−0.29	−2.84	0.005	−1.86 (1.20)	−4.21-0.49	−0.16	−1.55	0.121
**Daily internet usage time**										
4-6-<3	−1.01 (2.01)	−4.96-2.95	−0.05	−0.50	0.616	−0.32 (1.23)	−2.75-2.10	−0.03	−0.26	0.794
>7-<3	−3.33 (2.63)	−8.49-1.83	−0.17	−1.27	0.205	−1.86 (1.61)	−5.03-1.30	−0.16	−1.16	0.248
**Previous AI course**										
no-yes	−2.26 (2.50)	−7.17-2.65	−0.12	−0.91	0.365	0.02 (1.53)	−2.99-3.02	0.001	0.01	0.992
**Interest in AI technologies**										
no-yes	−2.45 (3.36)	−9.05-4.15	−0.13	−0.73	0.466	−1.37 (2.06)	−5.42-2.67	−0.12	−0.67	0.504
partial-yes	−1.41 (2.00)	−5.34-2.52	−0.07	−0.71	0.481	−1.23 (1.23)	−3.65-1.17	−0.11	−1.01	0.315
**Readiness to use AI**										
willing-not plan	2.80 (4.65)	−6.35-11.94	0.14	0.60	0.548	−1.88 (2.85)	−7.49-3.72	−0.16	−0.66	0.509
need training-not plan	6.67 (4.14)	−1.46-14.80	0.34	1.61	0.108	1.25 (2.54)	−3.74-6.23	0.11	0.49	0.623
use some system-not plan	10.41 (4.34)	1.87-18.94	0.53	2.39	0.017	3.31 (2.66)	−1.93-8.54	0.28	1.24	0.215
actively use-not plan	17.86 (4.74)	8.52-27.19	0.92	3.76	<0.001	7.60 (2.91)	1.87-13.33	0.66	2.61	0.009
**AI use purpose**										
daily information-academic	2.67 (2.03)	−1.32-6.66	0.14	1.31	0.189	1.71 (1.25)	−0.73-4.16	0.15	1.38	0.170
general use-academic	−3.15 (2.83)	−8.71-2.41	−0.16	−1.11	0.266	0.77 (1.74)	−2.63-4.18	0.07	0.44	0.654
never use-academic	−2.72 (3.77)	−10.13-4.69	−0.14	−0.72	0.471	1.10 (2.31)	−3.44-5.65	0.10	0.48	0.633
**AI replacing health care workers**										
no-yes	4.98 (2.74)	−0.40-10.35	0.25	1.82	0.070	1.39 (1.68)	−1.90-4.69	0.12	0.83	0.406
partly-yes	3.21 (2.79)	−2.28-8.71	0.16	1.15	0.251	3.82 (1.71)	0.45-7.19	0.33	2.22	0.026

The AILS (Cronbach’s α = 0.878) and MAIRS (Cronbach’s α = 0.972) demonstrated acceptable to excellent internal consistency, reflecting reliable assessments (Table 4). The mean total score for the MAIRS was 70.76 ± 19.40 (range: 22-110), which suggests a moderate to high perceived readiness among participants. The total AI literacy score averaged 59.84 ± 11.52 (range: 18-84).

**Table 4:** Scale scores on the Medical Artificial Intelligence Readiness Scale (MAIRS) and the Artificial Intelligence Literacy Scale (AILS)

Scales	Mean	SD	Min.	Max.	Skewness	Kurtosis	Cronbach’s alpha
Medical Artificial Intelligence Readiness	70.76	19.40	22	110	−0.389	0.001	0.972
Cognition	24.01	7.45	8	40	0.028	−0.199	0.947
Ability	26.62	7.89	8	40	−0.666	−0.214	0.955
Vision	9.91	3.04	3	15	−0.373	−0.314	0.902
Ethics	10.22	3.08	3	15	−0.627	−0.244	0.906
Artificial Intelligence Literacy	59.84	11.52	18	84	−0.259	0.537	0.878
Awareness	14.64	2.85	6	21	0.267	0.191	0.712
Usage	14.37	3.09	3	21	0.244	0.597	0.720
Evaluation	15.83	3.99	3	21	−0.968	0.674	0.930
Ethics	15.01	3.46	3	21	−0.131	−0.091	0.703

AI readiness was moderately and positively correlated with overall AI literacy (r = 0.338, *P* < 0.001). Among the subdimensions, Awareness (r = 0.384, *P* < 0.001) and Usage (r = 0.366, *P* < 0.001) had the strongest correlations with readiness (Table 5). Subscale analysis of MAIRS revealed that Cognition (r = 0.347, *P* < 0.001) and Ability (r = 0.298, *P* < 0.001) were most strongly related to AI literacy, especially to Awareness (Cognition r = 0.389, Ability r = 0.338; *P* < 0.001) and Usage (Cognition r = 0.385, Ability r = 0.316; *P* < 0.001). In contrast, correlations with Evaluation and Ethics were weaker across all dimensions.

**Table 5:** Correlations between the scores on Medical Artificial Intelligence Readiness Scale and Artificial Intelligence Literacy Scale and subscales

Scales	Artificial Intelligence Literacy	Awareness	Usage	Evaluation	Ethics
**Medical Artificial Intelligence Readiness Scale**	0.338	0.384	0.366	0.186	0.270
**Cognition**	0.347	0.389	0.385	0.207	0.252
**Ability**	0.298	0.338	0.316	0.157	0.254
**Vision**	0.261	0.311	0.307	0.133	0.188
**Ethics**	0.265	0.297	0.260	0.137	0.251

## Discussion

In this study, AI literacy was positively associated with medical AI readiness among health care students. In addition, medical students exhibited significantly higher AI readiness and AI literacy scores than nursing students. Medical and nursing students reported higher knowledge of AI in health care than respondents in earlier international studies (12-14). This discrepancy may be related to the lack of formal AI education in medical and nursing curricula (12), highlighting the need to integrate AI training into health care education. One possible explanation for this relatively higher self-reported knowledge is the increased visibility of AI tools in everyday life.

In previous studies, the proportion of nursing and medical students who had received training in AI varied from 4.7% to 20% (15-19). In the present study, this rate was 21.9% for medical and 10.8% for nursing students. Such variations may be attributed to geographic factors and differences in nursing and medical education curricula. Kwak et al revealed no significant association between having received AI training and the MAIRS score (17). Similarly, another study found that having attended prior AI-related courses did not significantly influence the attitudes of nurses and physicians toward AI (18). In contrast, several studies (2,19,20) reported that AI training was associated with higher readiness to use AI. The discrepancies between these studies may stem from variations in the quality, content, and duration of the AI training programs delivered across studies.

Several studies investigated AI readiness among both medical (2,15,20-23) and nursing students (15,20,24,25). In the present study, medical students demonstrated higher readiness levels than nursing students. This difference may be attributed to curricular and role-related distinctions between the two departments. Medical education typically provides greater exposure to diagnostic technologies and clinical decision-support systems, where AI is more directly applicable. Physicians are also expected to interpret complex data and make evidence-based decisions, further reinforcing the relevance of AI in their future practice. In contrast, nursing education tends to emphasize patient-centered care, empathy, and clinical application over technological competencies.

The lowest subdimension score in MAIRS was obtained on the Vision subscale, reflecting the students' knowledge of AI's limitations, strengths, and weaknesses. The highest score was obtained on the Ability subscale, which indicates that students have the potential to use medical AI effectively. This pattern aligns with previous research (5,20,21,25-27). Other studies have reported moderate (2,25,27-29) or low (2,22,24,30) levels of medical AI readiness among students. These lower scores reflect insufficient knowledge or a lack of preparedness to engage with AI technologies in clinical settings. Therefore, educational programs should integrate hands-on training to enhance students' readiness for the integration of AI into health care (24). Lower scores on the Ethics subscale were also observed in previous studies (2,10,21) and may indicate insufficient understanding of AI-related ethical issues, such as bias, data privacy, and accountability.

In the present study, male students exhibited significantly higher levels of medical AI readiness than their female counterparts, which was also reported elsewhere (16,20,22,27,31). However, some studies indicated no significant gender-based differences in AI readiness (2,5,18,26,28,32). These conflicting findings indicate that gender differences in attitudes toward AI can vary based on the specific context and population studied.

In this research, the study year was significantly associated with the MAIRS score. Second-year and third-year students demonstrated higher MAIRS scores than students in other academic years. This finding may be associated with recent exposure to foundational medical sciences and technology-related coursework. Consistent with this finding, Xuan et al reported that preclinical students scored higher on the Ability, Vision, and Ethics components of medical AI readiness compared with clinical students (2). However, a recent study did not find significant differences between the study year and the MAIRS score (27).

Students who had previously received education on AI demonstrated higher AILS scores than those without such training, which aligns with the results of other studies (33,34). Furthermore, students who actively used AI technologies exhibited higher AI literacy, a finding supported by previous research (32,34). Although several results were significant, most effect sizes were small, indicating that group differences were modest in practical terms. Regression analysis confirmed that study year, department, and AI readiness remained significant predictors of MAIRS. Students who were actively using AI and were open to development had notably higher scores. The only significant predictors for AILS were readiness to use AI and belief in AI’s partial role in replacing professionals. In this study, second-year, third-year, and fourth-year students had higher AILS scores than first-year students, which aligns with previous research (34). This suggests that increased exposure to clinical and technological contexts over the course of education may contribute to students’ developing AI-related competencies.

In this study, students with a strong interest in AI technologies and those willing to use or develop AI systems exhibited significantly higher levels of both AI readiness and literacy. These patterns were also supported in the adjusted model, where the readiness to use AI was the strongest and most consistent predictor for both outcomes. Moreover, students who perceived AI as having a valuable future role in health care demonstrated higher MAIRS and AILS scores, which reflects the influence of future-oriented thinking on AI acceptance. Interestingly, the highest AI literacy was found among those who believed that AI could partly replace health care professionals, which may indicate a more balanced and realistic perspective. Overall, these results emphasize the importance of interest, awareness, and critical thinking about AI to enhance readiness and literacy among health care students.

The reliability of the MAIRS in the present study (Cronbach’s α = 0.972) was higher than that of the original instrument (0.87) and the Persian version (0.94) (35). The internal consistency coefficient of the AILS was 0.878. The Cronbach’s alpha coefficients of recent studies were 0.85 (9), 0.814 (36), 0.83 (37), and 0.92 (38), which makes them similar to the original version (0.83) (8).

This study has several limitations. It was based on a convenience sample from a single institution. Therefore, the sample may not be fully representative of all medical and nursing students, and self-selection and nonresponse bias are possible. As a result, the generalizability of the findings to other settings may be limited. Given the number of bivariate comparisons conducted, the possibility of inflated type-1 error cannot be excluded. Therefore, bivariate findings should be interpreted cautiously and primarily as exploratory, with greater emphasis placed on the results of the multivariable regression analyses.

This study explored the relationship between AI literacy and readiness for medical AI among medical and nursing students, revealing moderate levels of both constructs and a positive association between them. Overall, the results highlight the educational importance of integrating structured, hands-on, and ethically grounded AI education into undergraduate health curricula to better align professional preparation with an increasingly AI-driven clinical landscape.
